# 1(OH) Vitamin D3 Supplementation Improves the Sensitivity of the Immune-Response during Peg-IFN/RBV Therapy in Chronic Hepatitis C Patients-Case Controlled Trial

**DOI:** 10.1371/journal.pone.0063672

**Published:** 2013-05-23

**Authors:** Yasuteru Kondo, Takanobu Kato, Osamu Kimura, Tomoaki Iwata, Masashi Ninomiya, Eiji Kakazu, Masahito Miura, Takehiro Akahane, Yutaka Miyazaki, Tomoo Kobayashi, Motoyasu Ishii, Norihiro Kisara, Kumiko Sasaki, Haruo Nakayama, Takehiko Igarashi, Noriyuki Obara, Yoshiyuki Ueno, Tatsuki Morosawa, Tooru Shimosegawa

**Affiliations:** 1 Division of Gastroenterology, Tohoku University Hospital, Sendai City, Japan; 2 Department of Virology II, National Institute of Infectious Diseases, Tokyo, Japan; 3 Department of Gastroenterology, South Miyagi Medical Center, Oogawara Town, Japan; 4 Department of Gastroenterology, Ishinomiki Red Cross Hospital, Ishinomaki City, Japan; 5 Department of Gastroenterology, Kosai Hospital, Sendai City, Japan; 6 Department of Gastroenterology, Rosai Hospital, Sendai City, Japan; 7 Department of Gastroenterology, Miyagi Shakai Hoken Hopital, Sendai City, Japan; 8 Department of Gastroenterology, Iwaki Kyoritsu Hosptal, Iwaki City, Japan; 9 Department of Gastroenterology, Oosaki Citizen Hoptial, Oosaki City, Japan; 10 Department of Gastroenterology, Iwate Central Hospital, Morioka City, Japan; 11 Department of Gastroenterology, Yamagata University School of Medicine, Yamagata City, Japan; Nihon University School of Medicine, Japan

## Abstract

**Objective:**

1,25(OH)_2_ vitamin D3 can affect immune cells. However, the mechanism responsible for the favorable effects of 1(OH) vitamin D3, which becomes 1,25(OH)_2_ vitamin D3 in the liver, is not clear. The aim of this study is to analyze the immunological response of 1(OH) vitamin D3 supplementation in CH-C patients.

**Design:**

Forty-two CH-C patients were treated with 1(OH) vitamin D3/Peg-IFNα/RBV. Forty-two case-matched controls were treated with Peg-IFNα/RBV. The expression of Interferon-stimulated genes (ISGs)-mRNA in the liver biopsy samples and JFH-1 replicating Huh-7 cells were quantified by real-time PCR. Ten kinds of cytokines in the plasma were quantified during treatment by using a suspension beads array. A trans-well co-culture system with peripheral blood mononuclear cells (PBMCs) and Huh-7 cells was used to analyze the effect of 1(OH) vitamin D3. The activities of the Th1 response were compared between subjects treated with 1(OH) vitamin D3/Peg-IFN/RBV and those treated with Peg-IFN/RBV therapy alone.

**Results:**

1(OH) vitamin D3/Peg-IFN/RBV treatment could induce rapid viral reduction, especially in *IL28B T/T* polymorphism. Several kinds of cytokines including IP-10 were significantly decreased after 4 weeks of 1(OH) vitamin D3 treatment (p<0.05). Th1 responses in the subjects treated with 1(OH) vitamin D3/Peg-IFN/RBV were significantly higher than those treated with Peg-IFN/RBV at 12 weeks after Peg-IFN/RBV therapy (p<0.05). The expression of ISGs in the patient’s liver biopsy samples was significantly lower than in those treated without 1(OH) vitamin D3 (p<0.05).

**Conclusion:**

1(OH) vitamin D3 could improve the sensitivity of Peg-IFN/RBV therapy on HCV-infected hepatocytes by reducing the IP-10 production from PBMCs and ISGs expression in the liver.

## Introduction

Hepatitis C Virus (HCV) is a non-cytopathic virus that causes chronic inflammation, fibrosis and hepatocellular carcinoma (HCC) [1]. Recently, it has been reported that vitamin D3 supplementation could improve the SVR in chronic hepatitis C (CH-C) patients [2], [3]. Moreover, the amount of 25-hydoxyvitamin D3 (25(OH) vitamin D3) in the serum could affect the treatment response to pegylated interferon α (Peg-IFN-α)/ribavirin (RBV) therapy and is complementary to interleukin 28B (*IL-28B*) rs1297860 C/T polymorphism in enhancing the correct prediction of the SVR in CH-C [4]. Another group reported that, in patients with genotype 1 HCV persistent infection, the 25(OH) vitamin D serum levels and *IL28B* polymorphism were independently associated with the likelihood of achieving a rapid viral response and SVR after treatment with Peg-IFN/RBV [5]. Although several kinds of mechanisms for the favorable effects of vitamin D3 supplementation were reported, the total effect of vitamin D3 supplementation remains unclear [6], [7]. One group reported that 25(OH) vitamin D3, but not vitamin D3 or 1, 25 dihydoxyvitamin D3 (1, 25(OH)_2_ vitamin D3), appeared to inhibit the viral life cycle at the level of infectious HCV assembly [7]. Another group reported that vitamin D3 or 1,25(OH)_2_ vitamin D3 and IFN-α could synergistically inhibit HCV production by enhancing the IFN signaling pathway [6]. However, the effect of vitamin D3 on the adaptive immune system in CH-C patients has not been reported yet.

It has been reported that vitamin D3, as synthesized in the skin by photolysis from 7-dehydrocholesterol or ingested with food, is transported in the blood to the liver where it is hydoxylated at the C-25-position [8]. Then, it is hydoxylated at the C-1α-position to form the active metabolite 1,25 (OH)_2_ vitamin D3 in the kidney [9], [10]. In this study, we selected 1(OH) vitamin D3, since the local concentration in the liver should be higher than other metabolites of vitamin D3. Moreover, 1 (OH) vitamin D3 is safe and commonly used in worldwide. 1,25 (OH)_2_ vitamin D3 is known to regulate calcium and phosphorus metabolism in skeletal homeostasis [11]. It has been reported that 1,25(OH)_2_ vitamin D3 plays an important role as an immune-modulator targeting various immune cells [12]–[15]. Various kinds of immune cells express not only vitamin D receptors (VDRs) but also vitamin D-activating enzymes, allowing local conversion of inactive vitamin D into 1,25 (OH)_2_ vitamin D3 within the immune system [16], [17]. The active metabolite1,25(OH)_2_ vitamin D3 could enhance the anti-mycobacterial activity in monocytes by enhancing the chemotactic and phagocytic capacity of macrophages [18]. Moreover, 1,25(OH)_2_ vitamin D3 might play an important role in the binding and capturing of antigens by dendritic cells (DCs) at the initiation of the immune response [19]. On the other hand, some groups reported that 1, 25(OH)_2_ vitamin D3 could inhibit the differentiation and maturation of DCs [19], [20]. In addition to monocyte-derived cells, CD3^+^ T cells, CD19^+^ B cells, natural killer cells (NK cells) could be directly and/or indirectly affected by 1, 25(OH)_2_ vitamin D3[12], [17], [21]–[24]. It has been reported that 1, 25(OH)_2_ vitamin D3 could contribute to the suppression of the immune response in autoimmune diseases [14], [15], [25]. More Recently, the expression of specific VDRs in liver cells and reduced expression of VDRs in CH-C patients have been reported [26]. In addition, an inverse relationship between the liver VDR expression and inflammation severity has been found [26]. However, the effects of 1, 25(OH)_2_ vitamin D3 for the adaptive immune system in the condition of CH-C patients and during treatment with peg-interferon α and ribavirin (Peg-IFN/RBV) are still unclear. Therefore, it is urgent to analyze the effect of 1, 25(OH)_2_ vitamin D3 on the adaptive immune responses that could contribute to the outcome of Peg-IFN/RBV therapy.

## Materials and Methods

### Study Design and Patients

**Figure 1:**
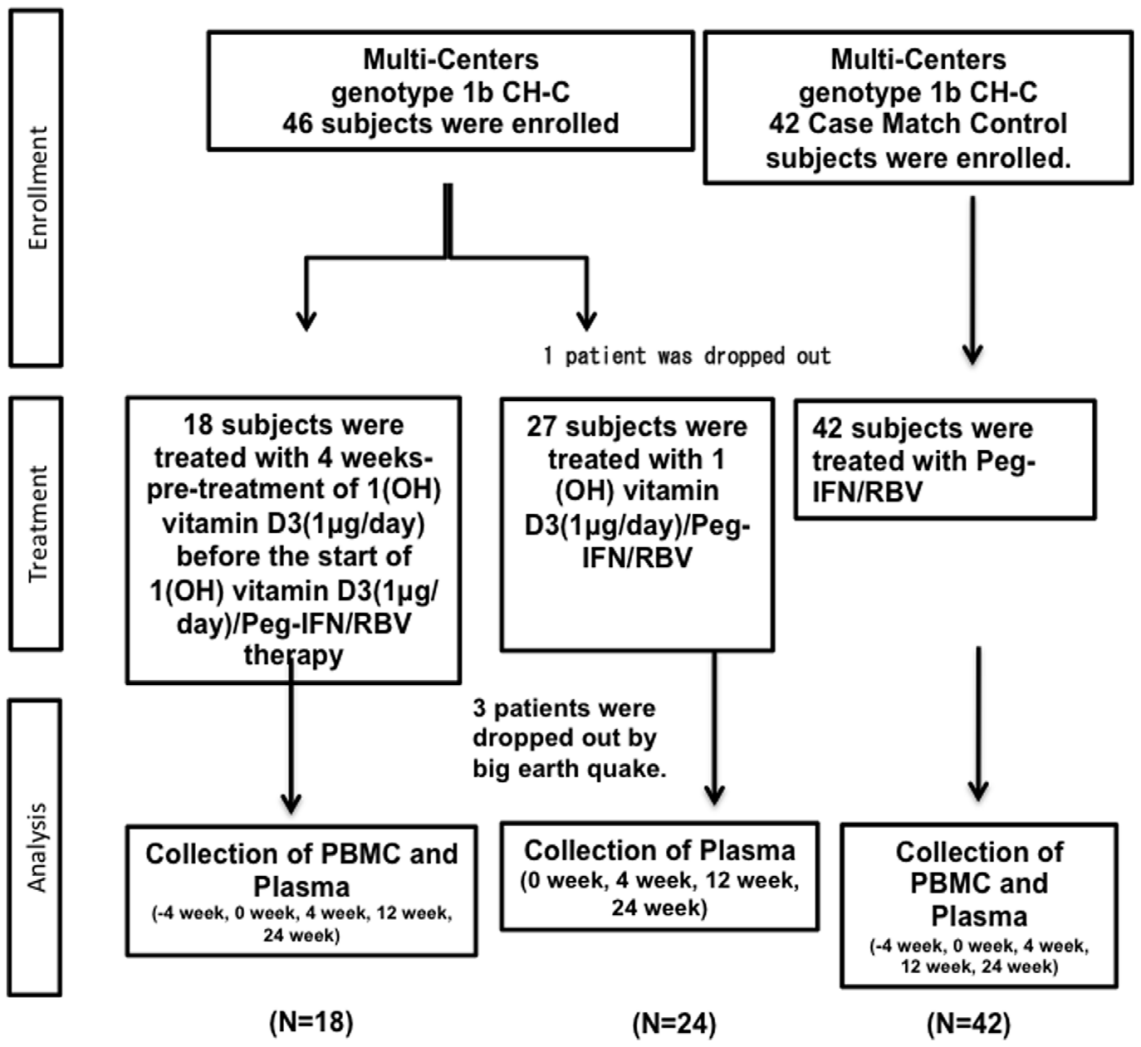
Enrollment of CH-C patients. 46 patients with genotype 1b and high viral loads were enrolled in this study. In total, 4 patients were dropped from this study.

Multi-centers that belong to the Tohoku-liver-study-group (TLG) were involved in this study. Dr. Abu-Mouch et al. reported that the SVR rate of Peg-IFN/RBV plus Vitamin D treatment group was 86% in the AASLD 2009 annual meeting [27]. On the other hand, the SVR rate of Peg-IFN/RBV treatment group was 42%. Considering the uncertainty, we speculated that the EVR rate might be 90% of the EVR rate in the Peg-IFN/RBV plus Vitamin D treatment group because the reported EVR rate in this study was remarkably high. Based on the results of this study, we enrolled about 80 patients including control patients: there was 10% loss in the proportion of patients during the 48 weeks therapy (α = 0.05, statistical power 90%) (EVR rate 77% vs 42%). The alfa level was two-sided. Forty-six CH-C (Genotype 1b) patients were enrolled in this study (Fig. 1). Forty-two matched historical controls treated with Peg-IFN-α/RBV therapy were analyzed. The inclusion criteria were as follows: age between 20 and 75 years, high viral load (>5.0 log copies/mL) by real time PCR analysis of HCV-RNA, absolute white blood cell count >2,000/ml, neutrophil count >1,000/ml, platelet count >90,000/ml, and hemoglobin concentration >11 g/dL in laboratory tests. The exclusion criteria were as follows: other liver diseases, including autoimmune hepatitis and alcoholic hepatitis, decompensated liver cirrhosis, liver failure, severe renal disorders, abnormal thyroid function, poorly controlled diabetes, poorly controlled hypertension, medication with immune-modulators, interstitial pneumonia and severe depression. Permission for the study was obtained from the Ethics Committee at Tohoku University Graduate School of Medicine (permission no. 2010-114) (UMIN000003694). The date of the protocol fixation was 10^th^ June 2010. The anticipated trial start date was 11^th^ June 2010. Patients in the 1(OH) vitamin D3/Peg-IFN/RBV group were treated from June 2010 to June 2012. Patients in the Peg-IFN/RBV group were treated from March 2009 to June 2012. Liver biopsy samples of the historical control were from previous studies (Permission no. 2009-166) (UMIN000002326), (Permission no. 2009-209), and (Permission no. 2010-404). Written informed consent of the control subjects treated with Peg-IFN/RBV treatment was obtained in the previous study and in the present study (Permission no. 2009-166) (UMIN000002326), (Permission no. 2009-209), and (Permission no. 2010-404). Written informed consent was obtained from all the participants enrolled in the 1(OH) vitamin D3/Peg-IFN/RBV treatment group. Participants were monitored for a year. At each assessment, patients were evaluated by hematological test, biochemical laboratory tests, immunological test and virological tests. Liver histology was analyzed at the start of Peg-IFN/RBV therapy using the METAVIR score.

### Detection of IL-28B Polymorphism

Genomic DNA was isolated from peripheral blood mononuclear cells (PBMCs) using an automated DNA isolation kit. Then, the polymorphism of *IL28B* (rs8099917) was analyzed using real-time polymerase chain reaction (PCR) (TaqMan SNP Genotyping Assay, Applied Biosystems, CA, USA). Detection of the *IL28B* polymorphism was approved by the Ethics Committee at Tohoku University Graduated School of Medicine (permission no. 2010-323).

### Isolation of Peripheral Blood Mononuclear Cells (PBMCs), CD4^+^ Cells and Cell Culture

PBMCs were isolated from fresh heparinized blood by means of Ficoll-Hypaque density gradient centrifugation (Amersham Bioscience, Uppsala, Sweden). Primary CD4^+^ cells were isolated using magnetic beads (Dynal). PBMCs were used to analyze the effect of the metabolite of α-calcidol(1(OH) vitamin D3) without direct cell to cell contact in an Huh-7 cells-transwell system. PBMCs and Huh-7 cells were cultured with serum-free complete medium that were previously made by our group [28]. A thousand times higher amount of 1(OH) vitamin D3 was used to analyze the effect of 1,25 (OH)_2_ vitamin D3, which comes from the lower part of chamber, since the Huh-7 cells have several enzymes that could convert 1(OH) vitamin D3 to 1,25 (OH)_2_ vitamin D3. The supernatant was harvested at 48 hours after the addition of 1(OH) vitamin D3 or 1,25 (OH)_2_ vitamin D3.

### Flow Cytometry Analysis

PBMCs were stained with CD3-pacific-blue, CD4-PE/Cy7, CD25-PE, CD127-APC, CD183 (CXCR3)-APC/Cy7, CD195 (CCR5)-FITC, Viaplobe and isotype control antibodies (BD pharmingen, San Jose, CA, USA) for 15 min on ice to analyze the frequency of CD3+CD4+CXCR3+CCR5+ cells (Th1) and CD3+CD4+CD25+CD127− (Tregs) by FACSCanto-II (BD). The FCS files 3.0 were analyzed by Flowjo 7.60 software.

### Multiplex Beads Suspension Array

The culture supernatant of PBMCs treated with the active vitamin D3 metabolite (1,25 (OH)_2_ vitamin D3) and the plasma obtained from CH-C patients treated with or without alfa-calcidol (1(OH) vitamin D3) were sequentially analyzed by suspension beads array (BIO-RAD Laboratories, Tokyo, Japan). Suspension beads array was performed following the manufacturer’s instruction. Briefly, the supernatant was incubated with first-antibody binding magnetic beads. Then, the detection antibody and PE conjugated streptavidin were reacted after the appropriate washing steps. Finally, the reaction plates were analyzed by Bio-plex 200 system.

### Real-time Polymerase Chain Reaction

RNA was isolated using a Qiagen RNeasy mini kit (Valencia, CA) and the yields were determined by absorption spectroscopy using a Nano-Drop (NanoDrop Products, Wilmington, DE). After the extraction of total RNA and the reverse transcription (RT) procedure, real-time polymerase chain reaction (PCR) using a TaqMan Chemistry System was carried out. The ready-made set of primers and probe for the amplification of IFN-γ, T-bet, Mx1 (ID Hs00895608), IFI44 (ID Hs00197427), IFIT1 (ID Hs01911452) and glyceraldehyde 3-phsphate-dehydrogenase (GAPDH) were purchased from Perkin-Elmer Applied Biosystems (Carlsbad, CA, USA). The relative amount of target mRNA was obtained by using the comparative threshold (CT) cycle method.

### The Quantification of ISGs mRNA in Hepatocyte Cell Culture

Huh-7 cells were treated with ethanol (control), 1(OH) vitamin D3 (1.0 µM) or 1,25(OH)2 vitamin D3 (1.0 µM) after transfection of poly IC (Sigma-Aldrich, St. Louis, MO) or in vitro transcribed JFH-1 full-length RNA. Cells were harvested 30 hour after transfection, and the expression levels of Mx, IFI44 and IFIT1 mRNA were assessed by real-time PCR using TaqMan Gene Expression Master Mix (Applied Biosystems, Carlsbad, CA) and gene-specific primer and probe sets (TaqMan Gene Expression Assay; Applied Biosystems) in accordance with the manufacturer’s instructions. The expression levels of genes with or without vitamin D3 treatment were expressed by the log fold increase of untreated Huh-7 cells.

### Statistical Analysis

**Figure 2:**
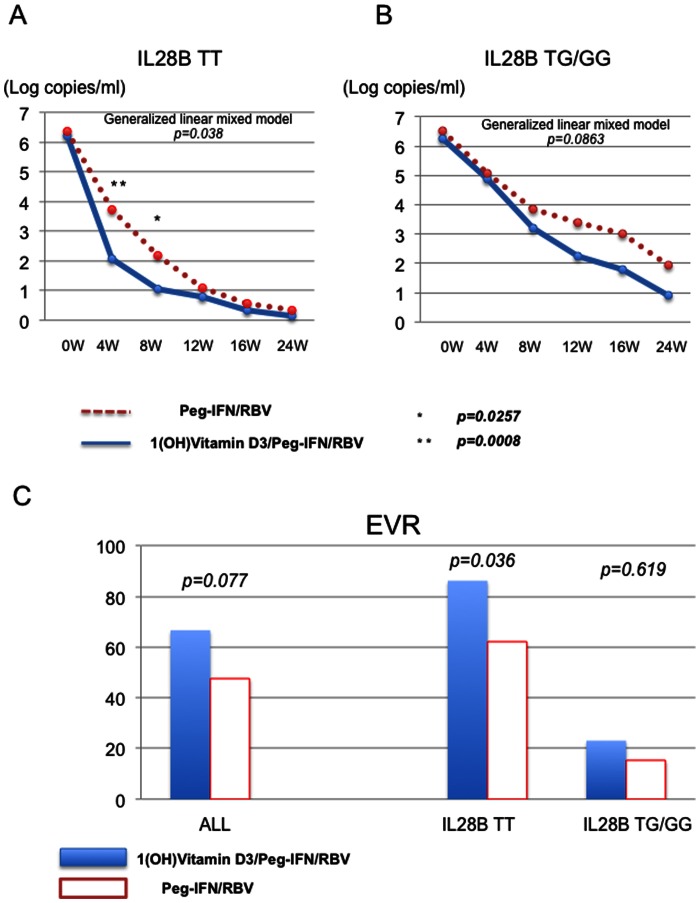
Comparison of viral dynamics and treatment response. Viral dynamics of subjects with IL28B T/T major homo polymorphism are shown (A). Viral dynamics of subjects with IL28B T/G or G/G minor polymorphism are shown (B). Blue lines indicate viral dynamics of subjects treated with 1(OH) Vitamin D3/Peg-IFN/RBV. Dotted lines indicate viral dynamics of subjects treated with Peg-IFN/RBV. *p<0.05 **p<0.01 The rates of early virological response in the patients treated with 1(OH) vitamin D3/Peg-IFN/RBV and Peg-IFN/RBV are shown (C).

**Figure 3:**
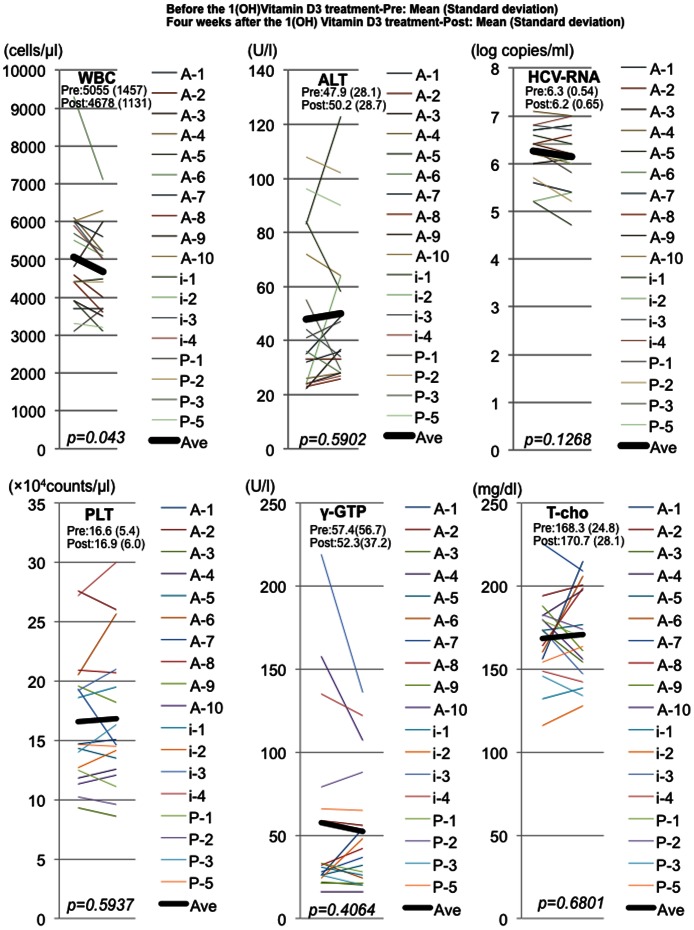
Comparison of hematological and biochemical analysis between before and after 4-week 1(OH) vitamin D3 treatment. Representative hematological, biochemical and virological data are shown. WBC indicates white blood cell count. ALT indicates alanine transaminase. HCV-RNA indicates titer of hepatits C virus RNA. PLT indicates platelet count. γ-GTP indicates gamma-glutamyl traspeptidase. T-cho indicates total cholesterol. The data at pre- and post-4weeks administration of 1(OH) vitamin D3 without Peg-IFN/RBV are shown. Black lines indicate the average of each analysis.

**Figure 4:**
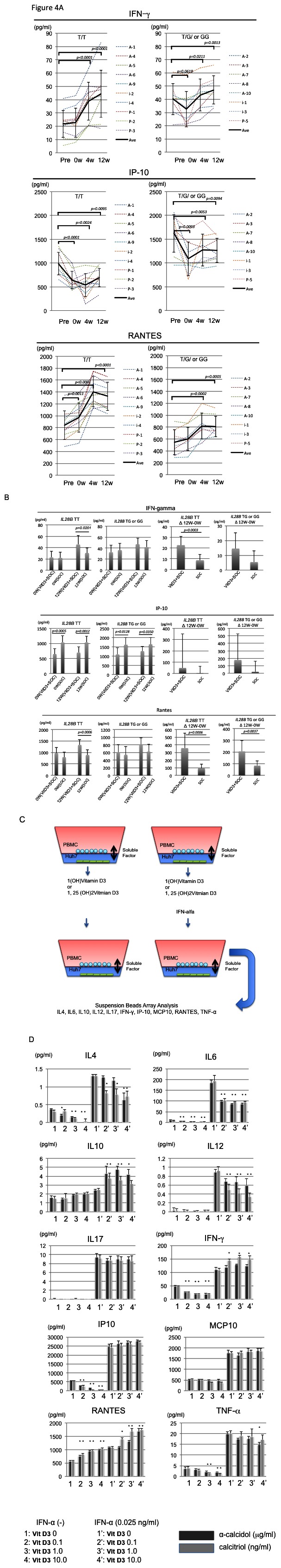
Cytokine profiles in the *ex vivo* and *in vitro* samples treated with vitamin D3. Sequential data of quantification of 3 cytokines (IFN-γ, IP-10 and RANTES) during 1(OH) vitamin D3 pre-treatment (pre vs 0w), 1(OH) vitamin D3/Peg-IFN/RBV therapy are shown (A). Dotted lines indicate the data of each subject. Black lines indicate the averaged data. Error bars indicate standard deviation. The data from IL28B (T/T) subjects or IL28B (T/G or G/G) subjects are shown in the independent graphs (A). Comparisons of the amounts of 3 cytokines (IFN-γ, IP-10 and RANTES) between the 1(OH) vitamin D3/PEG-IFN/RBV group (VitD3+standard of care (SOC)) and Peg-IFN/RBV group (SOC) at 0 weeks and 12 weeks after the start of Peg-IFN/RBV treatment are shown (B). Analysis of the changes in the amounts of the 3 cytokines (IFNγ, IP-10 and RANTES) during 12 weeks treatment of Peg-IFN/RBV is shown. Schema of *in vitro*-analysis of co-culture is shown (B). alfa-calcidol: 1(OH)vitamin D3 and calcitriol: 1,25(OH)vitamin D3 were used to analyze the cytokine production *in vitro*. Black bars indicate the data from samples treated with alfa-calcidol. Gray bars indicate the data from samples treated with calcitriol. *p<0.05.

**Figure 5:**
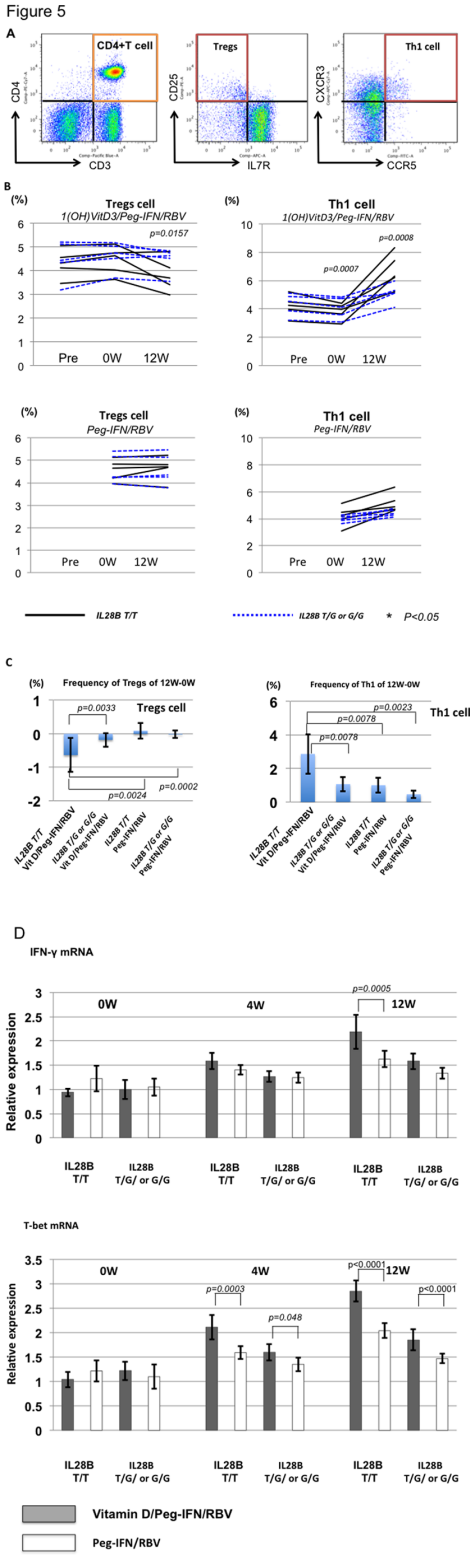
Comparison of Th1 and Tregs between 1(OH) vitamin D3/Peg-IFN/RBV and Peg-IFN/RBV. Representative dot plots of CD3^+^CD4^+^CD25^+^IL7R^−^ (Tregs) and CD3^+^CD4^+^CXCR3^+^CCR5^+^ (Th1 cells) are shown. (A) Frequencies of Th1 and Tregs among the 4 groups (IL28B T/T vitamin D3/Peg-IFN/RBV, IL28B T/G or G/G vitamin D3/Peg-IFN/RBV, IL28B T/T Peg-IFN/RBV, and IL28B T/G or G/G Peg-IFN/RBV) are shown. (B) Comparison of the T-bet and IFN-γ mRNA expression between subjects treated with vitamin D3/Peg-IFN/RBV therapy and those treated with Peg-IFN/RBV therapy. Each group included 5 patients. Total mRNA was extracted from isolated CD4^+^ T cells. The relative expression levels are shown in bar graphs. The statistical analysis was carried out by independent student *t*-test.

**Figure 6:**
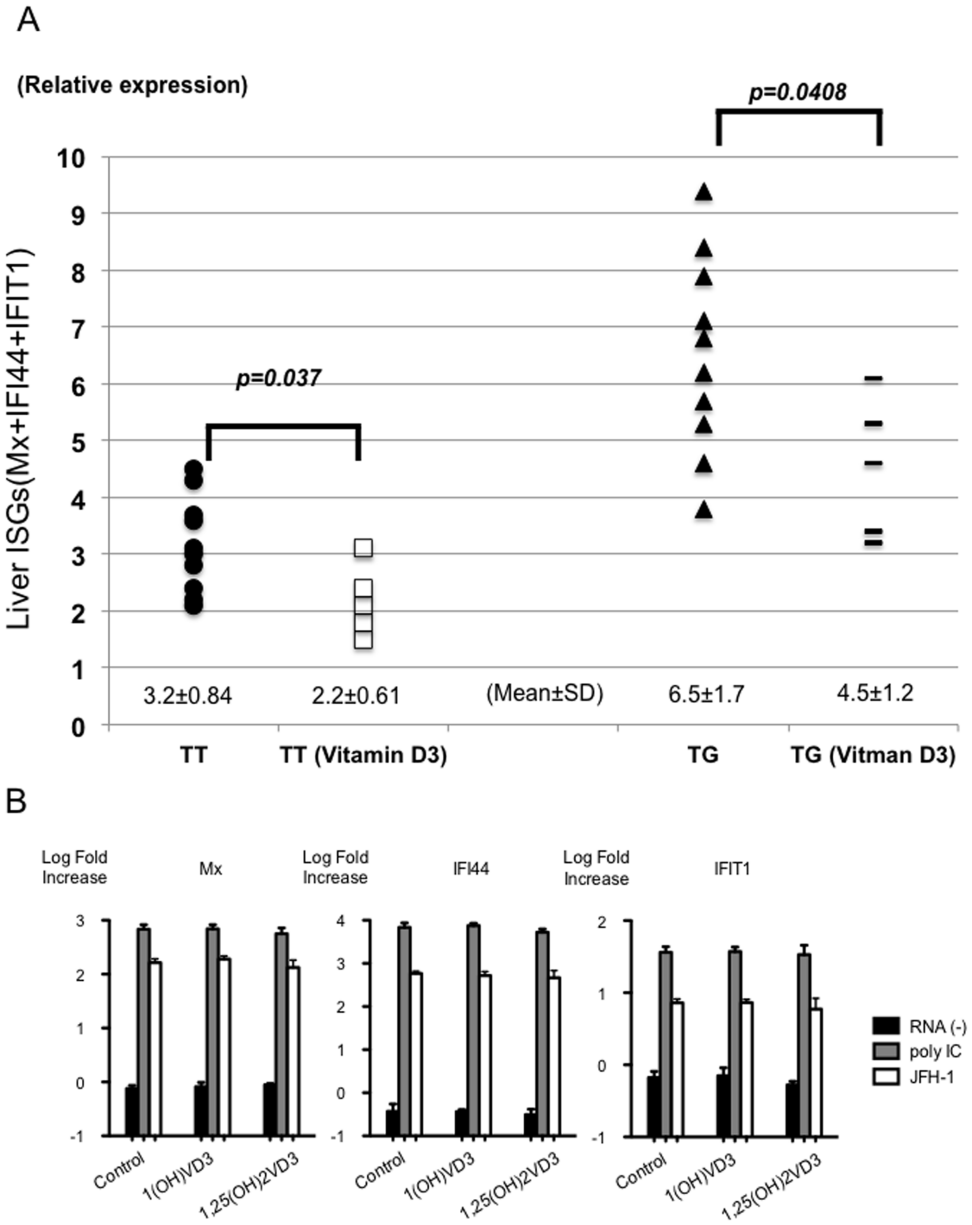
The effect of vitamin D3 on the expression of ISGs mRNA in the liver. The relative amount of target mRNA was obtained by using a comparative threshold cycle (CT) method. The expression levels of Mx, IFI44 or IFIT1 mRNA in an *IL28B T/T* patient treated without 1(OH) vitamin D3 are represented as 1.0 and the relative amounts of target mRNA in the other patients were calculated by the comparative Ct method [42]. Therefore, the standard amount of 3 ISGs (Mx, IFI44 and IFIT1) is 3. The relative amounts of the 3 kinds of ISGs were added and shown in the graph (A). Black circles indicate the data from IL28B (T/T) subjects treated without 1(OH) vitamin D3. White boxes indicate the data from IL28B (T/T) subjects treated with 1(OH) vitamin D3. Black triangles indicate the data from IL28B (T/G or G/G) subjects treated without 1(OH) vitamin D3. Black lines indicate the data from the subjects treated with 1(OH) vitamin D3 (A). The effect of vitamin D3 on the expression of ISGs mRNA in the hepatocyte cell culture are shown (B). Huh-7 cells were treated with ethanol (control), 1(OH) vitamin D3 (1.0 µM) or 1,25(OH)_2_ vitamin D3 (1.0 µM) after transfection of poly IC (Sigma-Aldrich, St. Louis, MO) or in vitro transcribed JFH-1 full-length RNA. Cells were harvested 30 h after transfection, and the expression levels of Mx, IFI44 and IFIT1 mRNA were assessed by real-time PCR using TaqMan Gene Expression Master Mix (Applied Biosystems, Carlsbad, CA) and gene-specific primer and probe sets (TaqMan Gene Expression Assay; Applied Biosystems) in accordance with the manufacturer’s instructions. The expression levels of genes with or without vitamin D3 treatment were expressed by log fold increase of untreated Huh-7 cells.

The data in Fig. 2A and B were analyzed using a generalized linear mixed model (Treatment group of 1(OH) vitamin D3/Peg-IFN/RBV and Peg-IFN/RBV were fixed-effect. Duration of treatment was random-effect.) and Student’s *t* test. The data in Fig. 2C were analyzed by χ^2^ test. The data in Fig. 3, Fig. 4A and Fig. 5B were analyzed by paired *t* test. The data in Fig. 4C were analyzed by Dunnett’s test. The data in Fig. 5C were analyzed by Tukey’s test. The data in Fig. 4B, Fig. 5D and Fig. 6 were analyzed by Student’s *t* test. The cut-off of acceptance of test’s results was *p*<0.05 with a confidence interval of 95%. All statistical analyses were carried out using JMP Pro version 10 (SAS Institute Inc., Cary, NC, USA).

## Results

### Efficacy and Tolerability of 1(OH) Vitamin D3 Combined with Peg-IFN/RBV Therapy

The characteristics of 42 patients treated with 1(OH) vitamin D3 (1 µg/day)/Peg-IFN/RBV therapy are shown in Table 1. The subjects enrolled in this study were 29 to 71 years old. 13 patients were previously treated with IFN-based therapy and failed to achieve SVR. Another 29 patients were treatment naïve. Case match control subjects treated with Peg-IFN/RBV therapy were enrolled in this study (Fig. 1) (Table 1). All of the enrolled patients had over 5 log copies/ml HCV-RNA and genotype 1b HCV RNA. Thirteen patients had the hetero/minor *IL28B* allele (T/G) (rs8099917) that was reported to be a marker of patients difficult-to-treat with Peg-IFN/RBV therapy [29]. Twenty-nine patients had the major homo *IL28B* allele (T/T) that was reported to be favorable for achieving SVR [29]. Therefore, we compared the viral dynamics between subjects treated with the 1(OH) vitamin D3/Peg-IFN/RBV and subjects receiving the Peg-IFN/RBV with the same IL28B polymorphism (Fig. 2A and B). The titers of HCV-RNA in the *IL28B* (T/T)-HCV patients treated with 1(OH) vitamin D3/Peg-IFN/RBV therapy were significantly lower than those treated with Peg-IFN/RBV at 4 weeks after the start of Peg-IFN/RBV therapy (*p<0.01*). The rate of early virological response in the *IL28B* (T/T) patients treated with 1(OH) vitamin D3/Peg-IFN/RBV was significantly higher than that in those treated with Peg-IFN/RBV alone (Fig. 2C). None of the patients showed side effects from 1(OH) vitamin D3 administration such as hypercalcemia or renal dysfunction, etc. The rate of the sustained virological response (SVR) in the overall patients treated with 1(OH) vitamin D3/Peg-IFN/RBV was 59.45% (45.24% in the overall patients treated with Peg-IFN/RBV) (*p = 0.2059*) The rate of SVR in the *IL28B* (T/T) patients treated with 1(OH) vitamin D3/Peg-IFN/RBV was 73.07% (55.17% in *IL28B* (T/T) patients treated with Peg-IFN/RBV) (*p = 0.1657*). However, this study was conducted to analyze the immunological response during the early phase of Peg-IFN/RBV. The sample size might not be large enough to analyze the SVR rate.

**Table 1:** Clinical characteristics of subjects enrolled in this study.

	PEG-IFNα/RBV	PEG-IFNα/RBV+VD3	PEG-IFNα/RBV+VD3	
	(n = 42)	(n = 42)	With Pre-VD3	Without Pre-VD3
			(n = 18)	(n = 24)
Gender(M/F)	19/23	15/27	6/12	9/15
Age	58.3(35–72)	59.1(29–71)	58.6(29–71)	58.5(43–71)
Body Weight	58.4	58.1(41.2–81)	56.4(41.2–81)	59.4(43–78)
History of IFN(+/−)	13/29	13/29	7/11	6/18
IL-28B(TT/TG,GG)	29/13	29/13	10/8	19/5
Sampling Point (week)	0W	All 0W	−4W	0W
HCV-RNA	6.3(5.1–7.2)	6.3(5.2–7.4)	6.3(5.2–7.1)	6.4(5.3–7.4)
ALT	68.5 (15–234)	66.4(16–242)	47.9(22–108)	78(16–242)
AST	55.2(16–161)	58.1(21–251)	45.3(22–112)	66.1(21–251)
WBC	5045(3050–7800)	5165(2400–9300)	5055(3100–9300)	5530(2400–8130)
RBC	441.3(355–522)	441.5(375–567)	450(375–567)	446(383–515)
PLT	16.6(9.4–29.4)	16.7(9.3–27.6)	16.6(9.3–27.6)	16.7(9.3–23.9)
Nue	2845(1750–5020)	2911(1190–7160)	2792(1190–7160)	3476(1533–5070)
Hb	13.8(11.8–15.9)	13.6(12–16.3)	13.7(12–15.2)	14.1(12.6–16.3)
Serum Ca	9.3(8.5–9.8)	9.2(8.6–10.1)	9.4(8.9–10.1)	9.2(8.6–10)
Insulin	9.4(6.8–20.2)	9.5(1.6–25.5)	9(4.76–20.8)	9.6(1.6–25.5)
T-cho	170.6(118–214)	172.4(116–227)	168.2(116–226)	173.7(119–227)
TG	108.5(55.6–210)	106.4(37–427)	118.9(37–259)	103.2(51–427)

HCV-RNA(log copies/ml), ALT(U/l), AST(U/l), WBC(counts/µl), RBC(x10^3^counts/µl), PLT(x10^4^counts/µl), Neut(counts/µl), Hb (g/dl), Serum Ca (mg/dl), Insulin (µU/ml), T-cho (mg/dl), TG (mg/dl).

### Biological Effect of 1(OH) Vitamin D3 Treatment during Peg-IFN/RBV Therapy

The biochemical and hematological analysis was carried out at 4 weeks before the start of Peg-IFN/RBV therapy and at the start of Peg-IFN/RBV therapy. Of those data, only the absolute counts of white blood cells were significantly decreased after 4 weeks-1(OH) vitamin D3-treatment (p<0.05) (Fig. 3). The titers of HCV-RNA were not significantly changed after the 4-week administration of 1(OH) vitamin D3 without Peg-IFN/RBV therapy. Therefore, we examined the immunological effects of 1(OH) vitamin D3. At first, we quantitated 10 cytokines (IL 4, IL 6, IL10, IL12, IL17, IFN-γ, IP-10, MCP-1, RANTES, TNF-α) in the peripheral blood samples during 1(OH) vitamin D3/Peg-IFN/RBV therapy using multiple beads suspension array (Fig. 4A and Fig. S1). Among the *IL28B* T/T polymorphism patients, the amounts of IL4, IP-10 and MCP1 in the peripheral blood serum were significantly reduced after 4-week-1(OH) vitamin D3-treatment. On the other hand, the amounts of IL6, RANTES and TNF-α in the serum were significantly increased after 4-week 1(OH)vitamin D3 treatment. In the *IL28B* T/G or G/G polymorphism patients, the amount of RANTES in the serum was significantly increased after 4-week 1(OH) vitamin D3-treatment. The amounts of IL4, IFN-γ, IP-10, MCP-1 in the serum were significantly decreased after 4-week 1(OH) vitamin D3-treatment. The administration of 1 (OH) vitamin D3 could reduce the high IP-10 status that is reported to be difficult-to-treat. Then, we compared the amounts of 10 cytokines between 1(OH) vitamin D3/Peg-IFN/RBV group and Peg-IFN/RBV group at 0 week and 12 weeks after the Peg-IFN/RBV treatment. The amounts of cytokines in the patients treated with 1(OH) vitamin D3/Peg-IFN/RBV at 0 week were affected by 4 weeks 1(OH) vitamin D3 pre-treatment. The amounts of IP-10 in the patients treated with 4 weeks-1(OH) vitamin D3 were significantly lower than those in the group treated without 1(OH) vitamin D3. However, the amounts of IFN-gamma and RANTES in the *IL28B* TT patients treated with 1(OH) vitamin D3/Peg-IFN/RBV were significantly higher than those in the *IL28B* TT patients treated with Peg-IFN/RBV without 1(OH) vitamin D3 at 12 weeks after the start of Peg-IFN/RBV treatment (Fig. 4B). In addition to the absolute amounts of several cytokines, the changes in the amounts after the 12 weeks Peg-IFN/RBV treatment were analyzed (Fig. 4B and Fig. S2). Changes in the amounts of IL4, IL-12, IFN-gamma and RANTES during the 12 weeks-treatment of Peg-IFN/RBV were significantly different between the 1(OH) vitamin D3/Peg-IFN/RBV group and Peg-IFN/RBV group (p<0.05) (Fig. 4B and Fig. S2).

### The Biological Effects of 1(OH)vitamin D3 and 1,25(OH)_2_ Vitamin D3 on the Production of Cytokines from PBMCs

Then, we examined whether the administration of 1(OH) vitamin D3 could affect the production of various kinds of cytokines from PBMCs. We used trans-well systems to analyze the effects of hepatocytes with various kinds of enzymes that affect the metabolism of 1(OH) vitamin D3 (Fig. 4C). We used a ng/ml order of calcitriol(1,25(OH)_2_ vitamin D3) as the active form of vitamin D3 and a µg/ml order of 1(OH) vitamin D3 as the pre-active form of vitamin D3 with or without IFN-α (0.025 ng/ml). The amounts of IL4, IL6, IFN-γ, IP-10 and TNF-α were significantly decreased by the active and pre-active form of vitamin D3 without IFN-α (Fig. 4D). Among them, the amount of IP-10 was dose-dependently decreased by 1(OH)vitamin D3 and 1,25(OH)_2_vitamin D3 without IFN-α. On the other hand, the amount of RANTES was dose-dependently increased by 1(OH)vitamin D3 and 1,25 (OH)_2_ vitamin D3 with or without IFN-α. The amounts of IL10 and IFN-γ were significantly increased by 1(OH)vitamin D3 and 1,25(OH)_2_vitamin D3 with IFN-α (Fig. 4D). These data indicated that 1(OH)vitamin D3 and 1,25(OH)_2_vitamin D3 could modulate the immunological status of PBMCs, especially the down-regulation of IP-10 production.

### Comparison of the Frequency of Th1 and Tregs between 1(OH) Vitamin D3/Peg-IFN/RBV and Peg-IFN/RBV

Sequential analyses of CD3^+^CD4^+^CXCR3^+^CCR5^+^(Th1 cells) and CD3^+^CD4^+^CD25^+^CD127^−^ (Tregs) were carried out during 1(OH) vitamin D3/Peg-IFN/RBV or Peg-IFN/RBV treatment. Representative dot plots indicating Th1 and Tregs are shown (Fig. 5A). The subsets of these cells could be clearly recognized by flow cytometry. Four-week treatment of 1(OH) vitamin D3 could significantly decrease the frequency of Th1 cells but not Tregs (p<0.05) (Fig. 5B). However, the frequency of Th1 cells was rapidly increased after the start of Peg-IFN/RBV therapy, especially in the *IL28B* T/T subjects treated with 1(OH) vitamin D3/Peg-IFN/RBV therapy (Fig. 5B and C). The frequency of Th1 cells in the subjects treated with 1(OH) vitamin D3 was significantly higher than in those treated with Peg-IFN/RBV at 12 weeks after the Peg-IFN/RBV therapy, especially in the *IL28B* T/T patients (Fig. 5C). Moreover, the expression of IFN-γ and T-bet mRNA in the isolated CD4^+^ cells of subjects treated with 1(OH) vitamin D3/Peg-IFN/RBV therapy was significantly higher than in those treated with Peg-IFN/RBV therapy at 4 weeks and 12 weeks after Peg-IFN/RBV therapy (Fig. 5D).

### Changes in ISG mRNA Expression in Liver with 1(OH) Vitamin D3 Treatment

The administration of 1(OH) vitamin D3 could reduce various kinds of cytokines in the serum. Therefore, we carried out quantification of ISG mRNA in samples from liver biopsies (Fig. 6A). We selected the Mx, IFI44, IFIT1 genes among the various kinds of ISGs, since another group previously reported that these ISGs could clearly recognize patients as difficult-to treat or easy-to-treat with IFN-based therapy [30]. The expression level of ISGs in the *IL28B* TT polymorphism was significantly lower than in the *IL28B* TG or GG polymorphism. Moreover, the expression levels of liver ISGs in the CH-C patients receiving 4 week-administration of 1(OH) vitamin D3 were significantly lower than those in the CHC patients without administration of 1(OH) vitamin D3.

### Direct Effect of Vitamin D on the Expression of ISGs in Hepatocyte without Immune Cells

We used Huh-7 cells with a JFH-1 system that mimicks the acute phase of ISG induction in HCV infection, since we wanted to determine whether 1 (OH) vitamin D3 and 1, 25 (OH)2 vitamin D3 could affect the ISG expression directly. Three representative ISGs (MxA, IFI44 and IFIT1) were analyzed by real-time PCR. JFH-1 replication could induce these ISGs in Huh-7 cells (Fig.6 B). We used 1(OH) vitamin D3 and 1,25(OH)_2_ vitamin D3 to analyze the ISG expression after JFH-1 inoculation. These ISGs were not affected by 1(OH) vitamin D3, and 1, 25(OH)_2_ vitamin D3 in vitro.

## Discussion

Recently, it has been reported that supplementation of vitamin D3, a potent immunomodulator, could improve the HCV response to antiviral therapy [2], [3], [31]. We used 1(OH) vitamin D3, since hepatocytes have various kinds of enzymes to convert 1(OH) vitamin D3 to the active metabolite 1,25(OH)_2_ vitamin D3. Therefore, we speculated that the administration of 1(OH) vitamin D3 could affect the liver adaptive immune cells since the local concentration of 1,25(OH)_2_ vitamin D3 might be higher than the systemic concentration of this active metabolite. Another group reported that 25(OH) vitamin D3, but not vitamin D3 or 1,25(OH)_2_ vitamin D3, could have direct-antiviral activity at the level of infectious virus assembly [7]. However, the antiviral activity of 25(OH) vitamin D3 is not so remarkable. Moreover, the system of HCV replication in that study did not include the immune cells that are important for the control of HCV replication [32]–[35].

In this study, we first reported that administration of 1(OH) vitamin D3 could affect the cytokine production from PBMCs and suppress the ISGs mRNA expression in the liver samples. Among the various kinds of cytokines, IP-10, which was reported to be an important biomarker for the treatment response, could be significantly decreased after 1(OH) vitamin D3 treatment in vivo [36], [37]. It has been reported that a high amount of IP-10 is a promising biomarker for difficult-to-treat patients regardless of the *IL28B* polymorphism [36], [37]. IP-10 can be produced from various kinds of immune cells including monocytes. In this study, we found that calcitriol could reduce the production of IP-10 from PBMCs dose-dependently *in vitro*. In addition to the production of IP-10, the expression of ISG mRNA in the liver biopsy samples with 1(OH) vitamin D3 treatment was significantly lower than in those without 1(OH) vitamin D3 treatment regardless of the *IL28B* polymorphism. The excessive expression of ISG mRNA before the Peg-IFN/RBV therapy might induce a poor response to IFN administration [38], [39]. In addition to these results, we confirmed that the amounts of IFN-gamma and RANTES induced by 12-weeks 1 (OH) vitamin D3/Peg-IFN/RBV treatment was significantly higher than those induced by 12 weeks Peg-IFN/RBV treatment without 1 (OH) vitamin D3. 1 (OH) vitamin D3 could suppress the basal levels of the immune response in the CH-C patients. However, the subsequent response of the adaptive immune system after the start of Peg-IFN/RBV treatment could have been augmented by 1(OH) vitamin D3. These data indicated that calcitriol might be able to stabilize the adaptive immune systems that were out of control in CH-C patients instead of inducing their activation. In this study, we could not detect a significantly higher rate of SVR in the 1(OH) vitamin D3/Peg-IFN/RBV group in comparison with those in the Peg-IFN/RBV group. However, the addition of 1(OH) vitamin D3 could improve the adaptive immune response. Therefore, the SVR rate in the 1(OH) vitamin D3/Peg-IFN/RBV group might have been significantly higher than in the Peg-IFN/RBV group, if the sample size had been large enough to analyze the SVR.

In addition to previous reports, our data indicated that calcitriol could affect the production of cytokines from PBMCs [25], [40]. However, we could not exclude the possibility of affecting cytokines other than the 10 cytokines we analyzed in this study. Moreover, other groups reported that vitamin D3 might modulate the expression of TLRs and/or their signaling, which are important in the immunopathogenesis of hepatitis C virus persistent infection [6], [14], [41]. This study was not a randomized control trial and did not have a large number of patients, since it focused on the effect of 1,25 (OH)_2_ Vitamin D3 on the immune cells. For this purpose, the number of included patients was sufficient for the analysis. Moreover, we are conducting a randomized control trial that includes a large number of chronic hepatitis C patients with sever fibrosis and low vitamin D3 concentrations (ongoing study) (UMIN000007400).

In conclusion, the active metabolite of vitamin D3, calcitriol, could improve the response to Peg-IFN/RBV therapy. Supplementation of 1(OH) vitamin D3 or 1,25(OH)_2_ vitamin D3 should be reasonable for the conditioning of IFN-based treatment including Direct Acting Antiviral (DAA)/Peg-IFN/RBV, DAA/Peg-IFN, Peg-IFN/RBV and Peg-IFN monotherapy.

## Supporting Information

Figure S1 **Cytokine profiles in the** ***ex vivo*** **treated with 1(OH) vitamin D3/Peg-IFN/RBV.** Sequential data of quantification of 7 cytokines (IL4, IL6, IL10, IL12, IL17, MCP-1 and TNF-α) during 1(OH) vitamin D3 pre-treatment (pre vs 0w), 1(OH) vitamin D3/Peg-IFN/RBV therapy are shown. Dotted lines indicate the data of each subject. Black lines indicate the averaged data. Error bars indicate standard deviation. The data from IL28B (T/T) subjects or IL28B (T/G or G/G) subjects are shown in the separate graphs.
(TIFF)

Figure S2 **Comparison of the cytokine profiles between 1(OH) vitamin D3 plus SOC and SOC.** Comparisons in the amounts of 7 cytokines (IL4, IL6, IL10, IL12, IL17, MCP-1 and TNF-α) between 1(OH) Vitamin D3/PEG-IFN/RBV group (VitD3+standard of care (SOC)) and Peg-IFN/RBV group (SOC) at 0 weeks and 12 weeks after the start of Peg-IFN/RBV treatment are shown. Analysis of the changes in the amounts of 7 cytokines (IL4, IL6, IL10, IL12, IL17, MCP-1 and TNF-α) during 12 weeks treatment of Peg-IFN/RBV is shown.
(TIFF)
