# Supplementary figures and images for: 1(OH) Vitamin D3 Supplementation Improves the Sensitivity of the Immune-Response during Peg-IFN/RBV Therapy in Chronic Hepatitis C Patients-Case Controlled Trial
Source: PLoS One. 2013 May 23;8(5):e63672. doi: 10.1371/journal.pone.0063672 (PMC3662784; doi:10.1371/journal.pone.0063672)

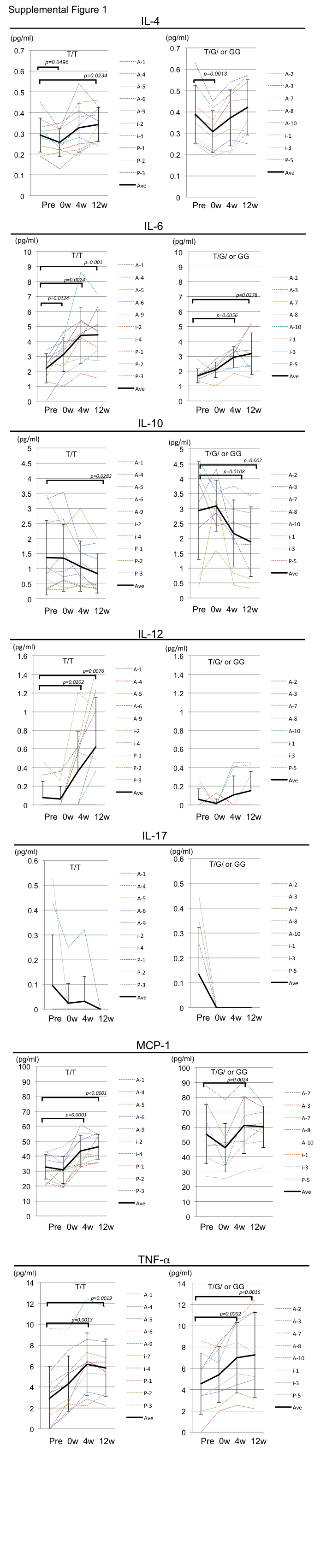

Supplement: Figure S1 — Cytokine profiles in the ex vivo treated with 1(OH) vitamin D3/Peg-IFN/RBV. Sequential data of quantification of 7 cytokines (IL4, IL6, IL10, IL12, IL17, MCP-1 and TNF-α) during 1(OH) vitamin D3 pre-treatment (pre vs 0w), 1(OH) vitamin D3/Peg-IFN/RBV therapy are shown. Dotted lines indicate the data of each subject. Black lines indicate the averaged data. Error bars indicate standard deviation. The data from IL28B (T/T) subjects or IL28B (T/G or G/G) subjects are shown in the separate graphs. (TIFF) [file pone.0063672.s001.tiff]
